# Seaweed-Derived Proteins and Peptides: Promising Marine Bioactives

**DOI:** 10.3390/antiox11010176

**Published:** 2022-01-17

**Authors:** Javier Echave, Paz Otero, Paula Garcia-Oliveira, Paulo E. S. Munekata, Mirian Pateiro, Jose M. Lorenzo, Jesus Simal-Gandara, Miguel A. Prieto

**Affiliations:** 1 Nutrition and Bromatology Group, Department of Analytical Chemistry and Food Science, Faculty of Food Science, Universidade de Vigo, 32004 Ourense, Spain; javier.echave@uvigo.es (J.E.); paz.otero@uvigo.es (P.O.); paula.garcia.oliveira@uvigo.es (P.G.-O.); 2 Centro de Investigação de Montanha (CIMO), Instituto Politécnico de Bragança, Campus de Santa Apolonia, 5300-253 Bragança, Portugal; 3 Centro Tecnológico de la Carne de Galicia, Avd. Galicia No. 4, Parque Tecnológico de Galicia, San Cibrao das Viñas, 32900 Ourense, Spain; paulosichetti@ceteca.net (P.E.S.M.); mirianpateiro@ceteca.net (M.P.); jmlorenzo@ceteca.net (J.M.L.); 4 Área de Tecnología de los Alimentos, Facultad de Ciencias de Ourense, Universidade de Vigo, 32004 Ourense, Spain

**Keywords:** seaweed, protein, peptides, bioactive, molecular mechanisms

## Abstract

Seaweeds are a typical food of East-Asian cuisine, to which are alleged several beneficial health effects have been attributed. Their availability and their nutritional and chemical composition have favored the increase in its consumption worldwide, as well as a focus of research due to their bioactive properties. In this regard, seaweed proteins are nutritionally valuable and comprise several specific enzymes, glycoproteins, cell wall-attached proteins, red algae phycobiliproteins, lectins, peptides, or mycosporine-like amino acids. This great extent of molecules has been reported to exert significant antioxidant, antimicrobial, anti-inflammatory, antihypertensive, antidiabetic, or antitumoral properties. Hence, knowledge on algae proteins and derived compounds have gained special interest for the potential nutraceutical, cosmetic or pharmaceutical industries based on these bioactivities. Although several molecular mechanisms of action on how these proteins and peptides exert biological activities have been described, many gaps in knowledge still need to be filled. Updating the current knowledge related to seaweed proteins and peptides is of interest to further asses their potential health benefits. This review addresses the characteristics of seaweed protein and protein-derived molecules, their natural occurrence, their studied bioactive properties, and their described potential mechanisms of action.

## 1. Introduction

Nowadays, the increase in pathologies related to diet such as cardiovascular diseases, obesity, or diabetes is a matter of concern in our society. Because of that, consumers are more interested in healthy natural products that could prevent the appearance of such diseases. In this sense, seaweed consumption is gaining importance in Western countries due to their good nutritional values. In addition, these organisms are a valuable source of bioactive compounds with increasing demand in food, pharmaceutical, and cosmetic applications [1]. In recent years, the protein content of seaweeds has been increasingly highlighted. These organisms contain high levels of essential amino acids (EAAs) and also specific proteins like lectins, glycoproteins (GPs), or phycobiliproteins (PBPs), which have been shown to exert different biological activities.

Table 1 shows the protein content and amino acids (AA) composition of some seaweed species often used as foods or food ingredients. It has been reported that protein content varies according to each taxonomic class, being green (Chlorophyceae), brown (Phaeophyceae), or red (Rhodophyceae) seaweeds [2]. According to data, protein concentration is generally higher in red seaweed species (12.5–35.2%), followed by green (9.6–23.3%), and brown (4.5–16.8%) (Table 1). Nonetheless, many red seaweed species have significant protein levels comparable to those found in fish, eggs, cereals, and soybean [3]. Considering this, protein extraction from seaweeds for functional applications can prove feasible [4].

AA composition is an important parameter to determine the nutritional quality of proteins, as higher EAA abundancies contribute to meeting their daily requirements [5]. Seaweed proteins (SPs) tend to contain high amounts of EAA, generally above 30% of their protein composition [6] but are also described to be above 40% (Table 1). This EAA abundancy is close to proteins and foods regarded as good sources of EAA, such as casein (43.6%), ovalbumin (52.4%), or legumes (~45%) [7,8]. Considering specific AA, most analyzed seaweeds show Glu and Asp following by Gly and Ala as the major AA [9,10]. In addition, seaweeds are rich in EAA like Val, Leu, Lys, and Phe, which are represented in high proportions from total AA (Table 1). It is noteworthy that monosodium glutamate, derived from Glu and naturally occurring in seaweed, is liable for the umami taste [11], and Gly and Ala are flavor-related amino acids that contribute to the particular taste of seaweeds [12]. Some seaweeds also seem to contain significant levels of Tau, which is a conditionally-EAA required for bile digestion but mainly found in meats [13].

**Table 1:** Protein content and aminoacidic composition of seaweeds.

Species	Protein (% dw)	AA Composition (g/100 g Protein)	Free AA (mg/g)	EAA (%TAA)	Ref.
Rhodophyceae					
*Palmaria palmata*	12.5	4.7 Thr, 6.1 Val, 3.6 Iso, 5.9 Leu, 0.5 Tyr, 3.8 Phe, 4.6 His, 5.6 Lys, 2.7 Met, 4.1 Cya, 3 Tau, 10.2 Asp, 5 Ser, 15.5 Glu, 5.8 Gly, 6.3 Ala, 2.1 Cys, 6 Arg, 4.4 Pro.	112.18	37.7	[14]
	16.2	7.6 Ala, 6.8 Arg, 12.5 Asp, 12.3 Glu, 6.5 Gly, 1.6 His, 4 Ile, 7 Leu, 7.7 Lys, 2.2 Met, 5 Phe, 5.5 Pro, 6 Ser, 5.3 Thr, 3.4 Tyr, 6.6 Val	124	24.8	[15]
*Porphyra* *dioica*	28.7	3.3 Asp, 3.1 Glu, 3 Ala, 2.3 Arg, 1.8 Gly, 1.6 Ser, 1 Tyr, 0.9 Pro, 1.1 Phe, 0.6 His, 1.1 Ile, 2.2 Leu, 2.2 Lys, 0.5 Met, 1.2 Thr, 1.2 Val	286.6	39.8	[3]
*Porphyra purpurea*	33.2	6.6 Asp, 4.6 Ser, 8.3 Glu, 7.5 Gly, 2.2 His, 9 Arg, 5 Thr, 8 Ala, 3.8 Pro, 1.3 Met, 0.4 Cys, 4.8 Val, 2.3 Lys, 3.4 Ile, 5.3 Leu, 7.8 Phe, 2.9 Tyr	n.a.	41.0	[16]
*Pyropia* *columbina*	24.6	12.2 Asp, 10.5 Glu, 6.1 Ser, 1.2 His, 8.8 Gly, 5.9 Thr, 6.1 Arg, 12.5 Ala, 3.9 Pro, 2.5 Tyr, 3.7 Phe, 5.8 Val, 1.6 Met, 1.9 Cys, 2.7 Ile, 0.6 Trp, 7.3 Leu, 6 Lys	n.a.	35.0	[10]
*Chondrus crispus*	35.2	5.5 Thr, 6.2 Val, 4.5 Ile, 6.9 Leu, 2.7 Tyr, 4.3 Phe, 2.1 His, 5.3 Lys, 3.3 Met, 2.9 Cya, 1.2 Tau, 12 Asp, 5.1 Ser, 12.1 Glu, 5.2 Gly, 7.5 Ala, 0.7 Cys, 6.5 Arg, 5.6 Pro	226.2	40.9	[14]
	19.5	3.6 Asp, 3.1 Glu, 4.6 Ser, 1.4 Thr, 1.8 His, 0.3 Gln, 0.3 Tau, 2.8 Arg, 0.4 Ala, 1.4 Tyr, 3.4 Lys, 2 Val, 0.2 Met, 1.5 Phe, 1.6 Ile, 2 Leu, 1.5 Hyp	72.8	46.7	[17]
*Osmundea pinnatifida*	24.3	4.8 Asp, 4.5 Glu, 3.9 Ser, 2.7 Thr, 3.7 His, 0.3 Gly, 0.1 Gln, 0.4 Tau, 2.4 Arg, 1.1 Ala, 1.7 Tyr, 3 Lys, 2.6 Val, 0.5 Met, 1.5 Phe, 2.1 Ile, 2.5 Leu, 1 Hyp	66.9	47.9	[17]
	20.7	13.6 Asp, 12.1 Glu, 2.7 Ser, 2.5 Gly, 0.9 His, 3.7 Arg, 5.7, Thr, 0.7 Ala, 15.8 Pro, 2 Tyr, 2.2 Val, 1.9 Met, 16.5 Ile, 2.2 Phe, 2.7 Lys	n.a.	41.6	[7]
*Gracilaria chilensis*	13.7	1.1 Asp, 1.5 Glu, 0.7 Ser, 1.1 His, 0.4 Gly, 0.6 Thr, 0.6 Arg, 0.6 Ala, 0.3 Tyr, 0.7 Val, 1.8 Met, 0.7 Cys, 0.8 Ile, 0.4 Leu, 1 Phe, 0.6 Lys	n.a.	42.8	[18]
*Gracilaria gracilis*	18.7	1.4 Arg, 0.1 His, 1.3 Lys, 1 Thr, 0.9 Ile, 1.2 Leu, 1 Val, 0.3 Met, 0.9 Phe, 0.9 Pro, 1.2 Ala, 0.6 Tyr, 2.1 Asp, 2.5 Glu, 1.3 Gly, 1.2 Ser	n.a.	45.6	[19]
*Gelidium corneum*	21	1.9 Ala, 0.8 Gly, 1.4 Val, 1.6 Leu, 0.9 Ile, 0.7 Thr, 0.8 Ser, 1.5 Pro, 2 Asp, 0.1 Met, 1.6 Glu, 1 Phe, 1.2 Lys, 0.3 His, 0.7 Tyr	n.a.	44.1	[20]
Phaeophyceae					
*Sargasum maclurei*	8.4	3.6 Thr, 6.2 Leu, 3.7 Ile, 4 Phe, 4.1 Lys, 1.3 Met, 2.1 Tyr, 2.3 Trp, 8.2 Asp, 29.7 Glu, 3.5 Cys, 1.3 His, 4.2 Gly, 3.9 Pro, 7.9 Ala, 3.8 Arg	74.9	27.8	[21]
*Fucus* *vesiculosus*	12.9	6.1 Thr, 5.8 Val, 2.1 Met, 5 Ile, 8.6 Leu, 5.4 Phe, 8 Lys, 1.9 His, 5.5 Arg, 3.2 Tyr, 16.7 Asn, 6.3 Ser, 19.7 Glu, 6.5 Gly, 9.8 Ala, 5.7 Pro, 2 Cys	119	40.9	[22]
*Fucus* *spiralis*	11.8	5.2 Arg, 7.2 Glu, 5.5 Ser, 2.7 Thr, 1.6 His, 0.1 Gln, 0.7 Tau, 1.5 Arg, 0.7 Ala, 3.7 Lys, 2.2 Val, 0.2 Met, 0,1 Trp, 1.2 Phe, 1,9 Ile, 1.8 Hyp	130	38.7	[17]
	9.7	5.6 Asp, 12.1 Glu, 11.4 Ser, 7.4 Gly, 3.2 His, 11.7 Arg, 10.8 Thr, 4 Ala, 6.9 Pro, 7.7 Tyr, 11.4 Val, 6.3 Met, 15.4 Leu, 15.3 Ile, 9.8 Phe, 12.5 Lys	n.a.	63.5	[7]
*Ascophylum nodosum*	4.5	6.9 Ala, 4.4 Arg, 16 Asp, 16.3 Glu, 5.9 Gly, 1.4 His, 4.4 Ile, 7.5 Leu, 5.5 Lys, 2 Met, 5.3 Phe, 4.5 Pro, 5.4 Ser, 5.8 Thr, 2.9 Tyr, 6 Val	35	29.2	[15]
	9.4	4.1 Asp, 7.2 Glu, 3.9 Ser, 1.9 Thr, 1.1 His, 0.7 Tau, 1.7 Arg, 1.5 Ala, 0.9 Tyr, 3.3 Lys, 1.9 Val, 0.4 Met, 0.1 Trp, 1.2 Phe, 1.6 Ile, 2.3 Leu, 1.6 Hyp	133	39.2	[17]
*Saccharina latissima*	12	11 Ala, 4.8 Arg, 13.4 Asp, 13.8 Glu, 5.6 Gly, 1.6 His, 4.4 Ile, 7.9 Leu, 5.9 Lys, 2.4 Met, 5.2 Phe, 4.5 Pro, 5 Ser, 5.3 Thr, 3.1 Tyr, 6 Val	98	32.7	[15]
*Bifurcaria bifurcata*	8.9	3.6 Thr, 3.7 Val, 1.7 Met, 2.9 Iso, 5.2 Leu, 3.3 Phe, 3.9 Lys, 1.3 His, 3.3 Arg, 1.7 Tyr, 8 Asn, 3.5 Ser, 15 Glu, 3.9 Gly, 8.4 Ala, 3.1 Pro	73.2	39.9	[22]
*Undaria* *pinnatifida*	16.5	4.3 Asp, 7.6 Glu, 5.8 Ser, 2.4 Thr, 1.4 His, 0.2 Gly, 0.6 Tau, 2.7 Arg, 3.4 Ala, 1.5 Tyr, 2.8 Lys, 2.5 Val, 0.7 Met, 1.7 Phe, 2 Ile, 3 Leu, 0.9 Hyp	64.7	37.2	[17]
	16.8	7.5 Asp, 4.1 Ser, 12 Glu, 6.5 Gly, 1.7 His, 8.8 Arg, 2.9 Thr, 9.7 Ala, 4.4 Pro, 0.1 Met, 0.3 Cys, 5.8 Val, 3.9 Lys, 5 Ile, 8.6 Leu, 4.8 Phe, 2 Tyr	n.a.	23.4	[16]
Chlorophyceae					
*Ulva* spp.	23.3	6.1 Asp, 5.2 Glu, 7.8 Ser, 2 Thr, 3.3 His, 0.2 Gly, 0.2 Gln, 0.3 Tau, 3.7Arg, 1.1 Ala, 1.9 Tyr, 3.2 Lys, 3.5 Val, 0.6 Met, 0.1 Trp, 2.4 Phe, 2.6 Ile, 3.5 Leu, 1.4 Hyp	116.2	43.6	[17]
*Ulva rigida*	17.4	3.1 Ile, 5.2 Leu, 3.7 Lys, 1.5 Met, 1.1 Cys, 3.3 Phe, 2.2 Tyr, 5 Thr, 5.6 Val, 1.4 His, 13 Asp, 9.4 Glu, 4.3 Pro, 6.1 Ser, 7.8 Gly, 12.3 Ala, 4.6 Arg	n.a.	30.8	[9]
	9.6	12.5 Asp, 9.4, 8.4 Ala, 6 Arg,6 Gly, 5.5 Ser, 3.2 Tyr, 4.4 Pro, 1 Hyp, 5.7 Phe, 2.9 His, 4.4 Ile, 7.8 Leu, 4.7 Lys, 1.3 Met, 4.8 Thr, o.4 Trp, 6.8 Val	4.9	40.8	[3]
*Ulva lactuca *	16.4	3.7 Ile, 6.7 Leu, 4.2 Lys, 1.6 Met, 0.4 Cys, 4 Phe, 2.1 Tyr, 4.7 Thr, 6.2 Val, 1.8 His, 12.3 Asp, 9 Glu, 5.3 Pro, 5.9 Ser, 10.7 Gly, 14.2 Ala, 3.6 Arg	n.a.	33.6	[9]
	15	8.4 Ala, 6.4 Arg, 12.1 Asp, 13.5 Glu, 6.4 Gly, 1.8, His, 4.2 Ile, 8 Leu, 5.5 Lys, 2.2 Met, 5.6 Phe, 4.7 Pro, 5.5 Ser, 5.5 Thr, 3.5 Tyr, 6.4 Val	106.1	36.9	[15]
*Ulva* *capensis*	17.3	3.5 Ile, 6-8 Leu, 3.7 Lys, 1.5 Met, 4 Phe, 2 Tyr, 5 Thr, 6.3 Val, 1.7 His, 17.2 Asp, 10.9 Glu, 3.6 Pro, 6.4 Ser, 8.8 Gly, 11.8 Ala, 3.3 Arg	n.a.	32.7	[9]
*Codium fragile*	10.8	0.8 Asp, 1 Glu, 0.5 Ser, 0.09 His, 0.5 Gly, 0.5 Thr, 0.4 Arg, 0.6 Ala, 0.3 Tyr, 1.4 Val, 0.9 Met, 0.1 Cys, 0.4 Ile, 0.7 Leu, 0.4 Phe, 0.5 Lys	n.a.	44.7	[18]
*Cladophora rupestris*	12	5.5 Ala, 6.5 Arg, 15.3 Asp, 15.3 Glu, 6.7 Gly, 1.4 His, 3.6 Ile, 7 Leu, 7.4 Lys, 1.8 Met, 4.5 Phe, 5.7 Pro, 4.3 Ser, 5.1 Thr, 4.3 Tyr, 5.8 Val	95.9	38.1	[15]

Notes and Abbreviations. AA: amino acids; EAA: Essential amino acids; TAA: Total amino acids; n.a.: Not analyzed. Amino acids: alanine (Ala); aspartic acid (Asp); arginine (Arg); asparagine (Asn); glutamic acid (Glu); glutamine (Gln); glycine (Gly); hydroxyproline (Hyp); histidine (His); isoleucine (Ile); leucine (Leu); lysine (Lys); methionine (Met); phenylalanine (Phe); proline (Pro); serine (Ser); taurine (Tau); tryptophan (Trp); tyrosine (Tyr); threonine (Thr); valine (Val).

Besides their value from a nutritional point of view, other SPs such as lectins, GPs, or PBPs have been reported to possess specific and relevant biological properties [23]. Mycosporine-like amino acids (MAAs) are other relevant aminic compounds in seaweeds account for similar biological properties. According to scientific literature, some of the bioactivities described for SP include antioxidant [24], anti-microbial and antiviral [25], antihypertensive [26], anti-inflammatory [27], anticancer, antithrombotic, and immunomodulatory properties [28]. All these effects depend on: (1) the chemical structure of the compounds, which differs among algae species, (2) parameters affecting seaweed biomass like season and location of collection (in general, the highest protein content in algae occur during the period between winter and early spring and the lowest from summer to early autumn), and (3) chemical alterations of the proteins during extraction and purification processes [15,17,28]. Furthermore, following a similar trend observed in other protein hydrolysates such as milk, ovalbumin, or soy hydrolysates, SP hydrolysates appear to contain derived bioactive peptides (BAPs) with a wide range of biological properties [29]. The development of these hydrolysates represents a challenge for scientists trying to identify the link between the chemical structure of peptides and their biological activities. However, numerous reports have evidenced their effective bioactive properties in vitro and in vivo. Indeed, in Japan, *Undaria pinnatifida* (wakame) and *Neopyropia yezoensis* (nori) digests have been approved as “foods for specified health uses” under claims of being hypotensive. Some of these products that include seaweed BAPs from these species are Wakame peptide jelly (Riken Vitamin Co., Ltd., Tokyo, Japan) or Nori peptide S (Shirako Co., Ltd., Tokyo, Japan) [30,31].

This work discusses the value and bioactive properties of SPs and derived BAPs. Their reported bioactive properties are highlighted with a special focus on described molecular mechanisms. Potential applications according to their specific bioactivities are explored, as well.

## 2. Seaweed Proteins and Derived Peptides

### 2.1. Glycoproteins

These compounds are proteins covalently linked to various oligosaccharide chains (glycans). Two major types of sugar chains are found in GPs, those bounded by N-glycosyl linkages or by O-glycosyl linkages [32]. GPs are located on the cell wall, on the surface of the cell, or free after secretion, and their roles include intercellular interactions and recognition [33]. Proportions of proteins and sugars of GPs differ from different algae species. For example, *Ulva* sp. GPs-rich fractions showed a protein proportion up to 33.4% [34], while in *Codium decorticatum,* which has GPs of molecular weight (MW) around 48 kDa, the protein proportion reached 60% [35]. Regarding the composition of the prosthetic fraction, seaweed GPs seem to mainly contain mannose [33]. Many seaweed GPs remain to be described, but other potential roles have been explored for bioactive properties in recent works, as shown in Section 3.

### 2.2. Lectins

Lectins are GPs that bind with high specificity to certain mono- or oligosaccharides. Seaweeds are good sources of novel lectins of low MW, especially red seaweeds [36]. The functions of lectins include gamete recognition and reproductive cell fusion, as well as defense against pathogens [36,37]. Lectins can be classified into four main groups: chitin-binding lectins, legume lectins, type-2 ribosome-inactivating proteins, and mannose-binding lectins [38]. In the case of seaweeds, the great majority of these proteins are mannose-binding, which are the major type of glycans found in them [33]. Likewise, relevant seaweed lectins are mannose-specific lectins, which show high binding affinity with these residues [39]. This property allows them to “agglutinate” particles containing these residues, as is the case of bacterial, viral, or eukaryote cell surface GPs [40]. One example is griffithsin, obtained from red *Griffithsia* sp. seaweeds, which has been described with diverse biological properties (Section 3) [41]. Due to these pharmacological properties, their characterization and isolation are a focus of research in medicine, molecular biology, or biochemistry. However, despite advances in the chemical characterization of seaweed lectins, additional information is still needed for a deeper understanding of their molecular structures, binding affinities, and possible biological functions for further applications [42].

### 2.3. Phycobiliproteins

**Figure 1:**
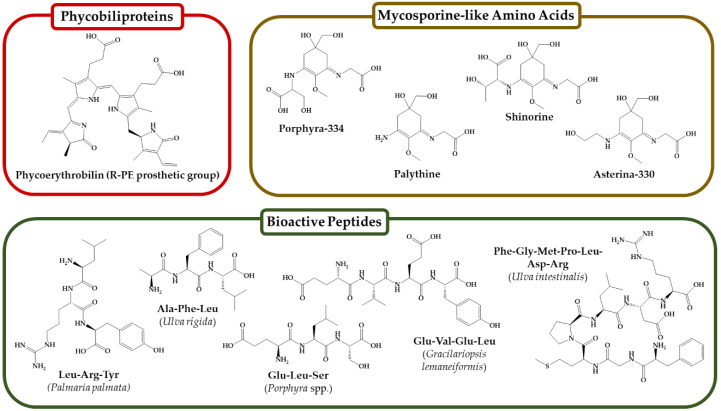
Chemical structure of the chromophore group of R-phycoerythrin, some relevant mycosporine-like amino acids, and bioactive peptides isolated from seaweed protein hydrolysates. Peptide sequences and source are presented in Table 3.

PBPs are hydrosoluble chromophore proteins mainly present in cyanobacteria but also in red seaweeds. Their primary metabolic role is to act as light absorbents during photosynthesis [43]. PBPs include four classes of pigments: blue colored phycocyanin with absorption maxima (λ_max_) in the range between 610 and 620 nm, magenta-colored phycoerythrocyanin (λ_max_: 575 nm), bluish-green-colored allophycocyanin (λ_max_: 652 nm), and deep red- or pink-colored phycoerythrin (PE) with λ_max_ between 540 and 570 nm [43]. The major PBP in red seaweeds is PE, of which phycoerythrobilin is its main prosthetic group (Figure 1) [44].

PBPs are used as natural pigments in food, replacing synthetic dyes, or as fluorescent probes in research [45]. Despite this, they are being studied for the development of nutraceuticals due to their bioactivities such as anti-viral, anti-cancer, antioxidant, and anti-inflammatory [46]. For this reason, current research on these molecules lies in function and biosynthesis mechanisms, structure elucidations, and potential applications. Isolation of PBPs has been reported in many species, for instance, *Neoporphyra haitanensis* [45], *Kappaphycus alvarezii* [47], *Centroceras clavulatum* [43], or *N. yezoensis* [47].

### 2.4. Mycosporine-Like Amino Acids

MAAs are small aminic secondary metabolites of MW below 400 Da (Figure 1) with strong absorption of ultraviolet (UV) radiation, typically between 320 and 360 nm. Thus, MAAs are useful molecules to protect cells from oxidative processes and solar UV-induced damage [48]. Up to now, more than 20 MAAs have been characterized in seaweeds and microalgae. From these, palaythine, asterina, shinorine, porphyra-334, mycosporine-glycine, usujirene, or palythene are most common in seaweeds [48]. They are naturally synthesized by various marine organisms, but seaweeds and microalgae are rich sources of them. However, they are much more abundant in red seaweeds, and only a small fraction of brown or green seaweeds presenting them [49]. For instance, porphyra-334 or shinorine are the most common in green or brown seaweeds such as *Ulva intestinalis* or *Alaria esculenta*, but red species like *Pyropia columbina* or *C. crispus* may additionally contain palythene much higher levels of mycosporine-glycine [49].

### 2.5. Protein-Derived Hydrolysates and Peptides

**Figure 2:**
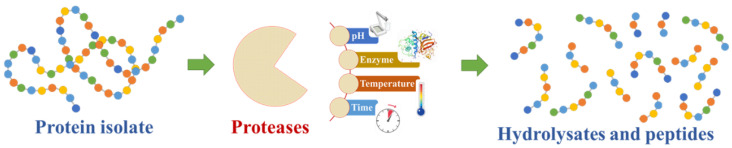
Schematic representation of seaweed protein hydrolysis into peptides. The hydrolyzation process usually involves the action of proteases and the adjustment of reaction parameters. Among the peptides produced by protein hydrolyzation, some display bioactive properties.

Peptides are protein fragments containing 2 to 40 AA in length generated from proteins by gastrointestinal digestion or from other hydrolyzation processes [50]. The importance of these molecules lies with the fact that some AA sequences, which are not active in SPs, show different biological properties after being released from the protein structure [51]. The development of algal peptides concentrates a blooming sector due to the diverse physiological activities of these molecules. Briefly, after the extraction and isolation of SPs, the main strategy to obtain BAPs from them involves their hydrolyzation (Figure 2). This may be performed by thermal, chemical, or enzymatic degradation, being the latter more extensively used since it allows more homogeneous and consistent patterns of hydrolyzation [23]. Thus, SP hydrolyzation using proteases can be used to maximize the yield of desired BAPs [23]. The process to describe the potential bioactive peptides requires testing different proteases in a single seaweed species [52,53,54], characterizing the BAP yield [55,56,57], optimizing the hydrolysis procedure [58], and exploring the effect of sequential hydrolysis using different proteases with defined conditions [59]. As hydrolyzation patterns and BAPs yielded are described, these approaches to define generated peptides allow covering a greater extent of potential bioactivities, as well as efficiency and effectiveness of extraction. Successful production of BPAs from hydrolyzed proteins of *Porphyra* spp, *Palmaria palmata*, *Ulva* spp, among others, have been reported [60,61,62].

## 3. Bioactive Properties and Mechanisms of Action

**Figure 3:**
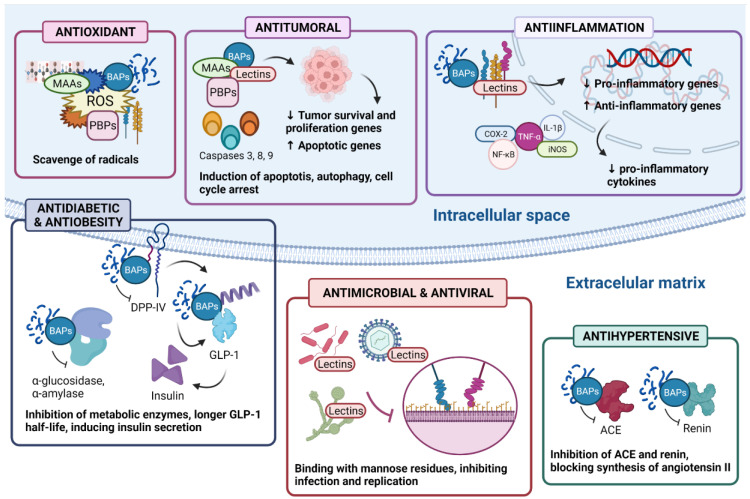
Summary of reported mechanisms of action of several SPs and BAPs. Their location with respect to the bilayer membrane represents that these effects are inducted inside, outside the cell, or on the cell surface. Abbreviations: PBPs: phycobiliproteins; MAAs: mycosporine-like amino acids; BAPs: bioactive peptides; ROS: reactive oxygen species; IL-1β: interleukin-1β; TNF-α: tumor necrosis factor- α; COX-2: cyclooxygenase-2; iNOS: inducible nitric oxide synthase; NF-κB: nuclear factor- κB; DPP-IV: dipeptidyl peptidase-IV; GLP-1: glucagon-like peptide-1; ACE: angiotensin I-converter enzyme.

As described, a wide number of bioactive properties have been reported for SPs, derived hydrolysates, and peptides. However, as their study is still recent, many species and synthesized hydrolysates are yet to be further studied, as well as their possible mechanisms of action. As they are accounted for higher protein content, red algae have been the focus of research in this matter, making them more represented in literature than other groups. Nevertheless, some studies detail or elucidate these mechanisms based on their results. Figure 3 depicts a summary of the described mechanisms of action reported for SPs, BAPs, and MAAs.

### 3.1. Antioxidants

Oxidative stress is considered the major cause liable for a number of hazardous effects on health, such as inflammation, cell death, or unregulated cell proliferation, which in turn, may result in many ailments and diseases such as chronic inflammation, metabolic disorders, or ultimately, cancer [63]. It is mainly caused by the interaction of overabundant reactive oxygen species (ROS) with cell components such as membrane lipids, proteins, or DNA, among others, which leads to the disruption of normal cell metabolism. The ability of antioxidants to “quench” these ROS and avoid potential cell damage ameliorates these hazardous effects [64]. This property is intrinsically related to the chemical structure of antioxidants. In the case of SP and BAPs, their antioxidant activity may be related to their amino acid composition and conformation [40].

Several studies have corroborated the antioxidant properties of SPs. For example, the antioxidant activity of a purified GP from *C. decorticatum* was assessed based on the ability to scavenge 2,2-diphenyl-1-picrylhydrazyl (DPPH), hydroxyl, superoxide, and nitric oxide radicals. According to the results, the GP showed positive results in a dose-dependent manner, which was attributed to the presence of aromatic amino acid present in its chemical structure [35,65]. A thyroglobulin-binding lectin from *Sargassum fusiforme* inhibited up to 80% of DPPH radicals and 60% of 2,2′-azino-bis(3-ethylbenzothiazoline-6-sulfonic acid) (ABTS) radicals. Authors considered that these results were due to its glycan and aminoacidic composition, rich in G, T, and A [40]. Regarding MAAs, these compounds have been long-known to be effective antioxidants, especially against UV-light induced oxidation [66]. A recent and extensive study evaluated the antioxidant activity of a wide number of MAAs isolated from *G. domingensis*, such as asterina-330, shinorine, palythine, or palythinol [24]. Among the isolated MAAs, asterina-330 showed by far the most significant antioxidant activity with 10.03 μmol Trolox equivalents/mol of the compound, in contrast with the 2953 μmol Trolox equivalents/mol of the compound obtained for gallic acid after 1 h of ABTS assay.

Numerous studies have successfully produced BAPs with antioxidant properties for different seaweed species. According to data, it has been suggested that BAPs display higher antioxidant activity than SPs or SP hydrolysates of higher MW. This could be due to the enhanced cell and chemical accessibility of released amino acids from proteins. For example, fractionation of *P. palmata* chymotrypsin-produced BAPs based on their MW showed that those fractions with lower ones displayed higher antioxidant activities. The fraction containing peptides <10 kDa displayed the most significant antioxidant properties following a DPPH assay [55]. In another study conducted with the same species, the authors obtained a peptide SDIAPGGNM, which showed an oxygen radical absorbance capacity (ORAC) of 152 nmol TE/μmol peptide and a ferric reducing antioxidant power (FRAP) of 21 nmol TE/μmol peptide [67]. Other seaweeds have also been reported to be a source of BAPs. For example, proteins from *P. haitanensis* were digested using pepsin, and their antioxidant properties were evaluated in vitro by 2,2-diphenyl-1-picrylhydrazyl (DPPH) assay and ferric reducing antioxidant power (FRAP), showing positive results [68]. Pepsin and pepsin-Corolase^®^ hydrolyzed biomass from *P. dioica* resulted in a diverse BAP pattern, of which the pepsin fraction displayed significantly higher antioxidant activity on various antioxidant assays. Yet both treatments resulted in an increase in antioxidant activity, which was related to the release of >10 kDa peptides [69]. In another study by the same authors, *P. dioica* biomass and its protein isolate were hydrolyzed using the proteases Prolyve^®^ and Flavourzyme^®^. This digestion achieved a 5-fold increase in antioxidant activity. The authors noted that phenolic compounds were also released after enzymatic digestion, which paired with a positive correlation with protein degradation, suggesting a synergistic dynamic between these elements [70]. Finally, a recent study characterized the optimum hydrolysis conditions of *G. lemaneiformis* proteins with chymotrypsin [58]. The variables considered in this experiment were enzyme/substrate ratio (1–3%), temperature (41–19 °C), and pH (8.5–9.5), whereas substrate concentration and reaction time were kept constant (10 mg/mL and 2.0 h, respectively). According to the authors, the optimum conditions to improve the extraction of antioxidant peptides were enzyme/substrate ratio (1:10), 46.4 °C, and pH 9.2. After separation and purification steps, GLWKTF was identified as the principal antioxidant, whose properties were attributed to its small molecular size and the presence of hydrophobic and/or aromatic amino acids [58].

### 3.2. Antimicrobials and Antivirals

Among SPs, lectins have been reported in recent years to be potent and effective antibacterial and antiviral agents due to their binding with monosaccharide residues of cell walls or membrane of yeasts, bacteria, and viruses [25,36]. Specifically, different mannose-binding lectins have been isolated from seaweeds, which have potent antiviral properties, as many viruses present mannose residues in their capsid or envelope [36]. For example, two recently isolated lectins from the green alga *C. isthmocladum* with galactose-binding activity were found to inhibit biofilm formation of *Staphylococcus aureus* and *S. epidermidis* up to 60%, compared to the control samples (Table 2). However, they did not display any significant antibacterial or antibiofilm activity against *Escherichia coli* [71]. The mannose-binding lectin griffithsin, derived from the red macroalga genus *Griffithsia* sp., has become a main focus of research since it was isolated in 2004 [72]. Griffithsin has been thoroughly studied for its potential applications against human and cattle viral infections, showing promising results against human immunodeficiency virus (HIV), human herpes virus (HSV), hepatitis C or severe acute respiratory syndrome-coronavirus (SARS-CoV), among others, both in vitro and in vivo [73,74]. Indeed, recent work has demonstrated that the synergistic cutaneous application of carrageenan and griffithsin inhibited simian-HIV in rhesus macaques, as well as HSV and HPV in mice [75]. The broad action range of griffithsin against viruses with diverse structural features has made it to be proposed as an antiviral agent against SARS-CoV-2, as it can effectively bind to the Spike surface GPs of SARS-CoV, inhibiting cell-to-cell infection [76]. It has also been shown to have a prolonged half-life, both by oral and parenteral administration, which supports its potential application in antiviral treatment [77]. Grifonin-1, a griffithsin-derived peptide, has also shown significant inhibition of HIV infection in in vitro cultures, along with a very low cytotoxicity index [78]. In addition, a mannose-binding lectin recently isolated from *Grateulopia chiangii* showed significant inhibition against various strains of influenza, like H1N1, and towards HSV1 and HSV2 (Table 2) [79]. Nevertheless, it did not show antiviral activity against HIV, indicating that although broad, the action range of these lectins is dependent on their binding site affinities.

Apart from lectins, to our knowledge, there are few reports on antimicrobial activity from other SP and BAPs. For example, a study assessed the antifungal properties of PBPs from *Hydropuntia cornea* against the common rotting fungi, *Botrytis cinerea*, assessing its potential growth inhibition when applied to tomatoes. With a concentration of 0.3 mg/mL, PBPs successfully inhibited fungal growth and spore germination by 33% and 80%, respectively [80]. According to the literature, it could be suggested that other protein-derived components could act as antimicrobial agents, but especially in combination with other seaweed components like phenolics and sulfated polysaccharides. In the study of Beaulieu et al., a hydrolysate from *S. longicruris* was obtained using trypsin, and antimicrobial properties were tested. The hydrolysate inhibited up to 40% of the growth inhibition on *S. aureus*. However, when individual peptides were isolated, no antimicrobial activity was observed. Authors proposed that this could be due to the co-extraction of sulfated polysaccharides, which are indeed accounted for antimicrobial properties [81].

**Table 2:** Reported bioactive proteins from seaweeds and their mechanisms of action.

Species	Protein(s)	Bioactivity	Mechanism of Action	Ref.
Rhodophyceae				
*Griffithsia* sp.	Lectin (Griffithsin)	In vivo, antiviral	Mannose-binding lectin binds with viral envelope GPs, inhibiting viral infection of HIV-1, HSV, and HPV	[75]
		In vitro, antiviral	Mannose-binding lectin binds with Spike GPs, inhibiting viral infection of SARS-CoV	[82]
		In vitro, antiviral	Griffithsin and synthetic polymers showed high virucidal activity in cell-associated HIV-1 and in the presence of seminal and vaginal simulants. Blocking of CD4+ viral binding	[83]
*Grateloupia chiangii*	Lectin	In vitro, antiviral	Mannose-binding lectin binds with viral envelope GPs, inhibiting viral infection of Influenza H1N1 and HSV1/2	[79]
*Kappaphyrus alvarezii*	Lectin	In vitro, antiviral	Mannose-binding lectin binds to Influenza hemagglutinin and HIV GP gp120, avoiding infection	[84]
*Kappaphycus striatum*	Lectin	In vitro, antimicrobial	Growth inhibition of *Vibrio alginolyticus* (25.4 μg/mL) and *Enterococcus cloacae* (101.6 μg/mL). Did not inhibit other *Vibrio* spp., *Staphylococcus aureus*, or *Escherichia coli*	[85]
*Gracilaria fisheri*	Lectin	In vivo, antimicrobial	There was >50% mortality reduction in 100 μg/mL treated shrimp, related with agglutination of *Vibrio parahaemolyticus*	[25]
*Hydropuntia cornea*	PBPs	In vitro, antifungal	Dosage of 0.3 mg/mL inhibited *Botrytis cinerea* growth (33%) and spore germination (80%)	[80]
*Neopyropia yezoensis*	GP	In vitro, anti-inflammatory	Inhibition of TLR4 and ERK1/2 activation, inhibition of NF-κB release	[86]
*Amansia multifida*	Lectin	In vivo, anti-inflammatory	Paw edema reduction against several pro-inflammatory agents. Reduction in TNF-α, IL-1β levels, and neutrophil migration. Increased glutathione levels	[87]
*Eucheuma cottonii*	Lectin-rich extract	In vivo, anti-inflammatory	Decreased mucin synthesis, leucocyte infiltration, and TNF-α, NF-κB, IL-4, MMP-9, EGFR levels in asthmatic rats	[88]
*Briothamniun triquetum*	Lectin	In vivo, anti-inflammatory	Reduced peritonitis and paw edema in treated mice. Reduction in TNF-α, IL-1β, MPO levels, and neutrophil migration. Better results than the positive control	[42]
*Soliera filiformis*	Lectin	In vitro, antitumor	Apoptosis induction. Bcl-2 downregulation, upregulation of caspases 3,8,9	[89]
*Gracilaria lemaneiformis*	PE	In vitro, antitumor	Apoptosis induction. Increased caspase 3/9 and p53 expression	[90]
*Portieria hornemannii*	R-PE	In vitro, antitumor	Apoptosis induction. Cell cycle arrest at G_2_/M phase, membrane blebbing, and >80% cell deaths at 1 mg/mL	[91]
*Eucheuma serra*	Lectin	In vitro, antitumor	Apoptosis induction. Increased caspase-3 expression, binding to mannose in human sarcoma cells. Complete cancer cell death at 2 μg/mL, but not in normal cells	[92]
*Bryothamnion seaforthii*	Lectin	In vivo, wound healing	Induced greater inflammatory response, fibroblast proliferation, and collagen synthesis. Reduced wound size and faster closure in treated mice	[93]
Phaeophyceae				
*Sargassum fusiforme*	Lectin	In vitro, antioxidant	Dosage of 1.6 mg/mL inhibited 77.23% DPPH; 4 mg/mL inhibited 68.97% ABTS	[40]
*Saccharina japonica*	GP	In vitro, antioxidant	DPPH (IC_50_ = 0.12 mg/mL), ABTS (IC_50_ = 0.05 mg/mL), FRAP (IC_50_ = 0.3 mg/mL) and xanthine oxidase (70%, 0.2 mg/mL) inhibition	[94]
*Undaria pinnatifida*	GP	In vitro, antidiabetic	Rat intestinal (IC_50_ = 0.29 mg/mL) and yeast (IC_50_ = 0.11 mg/mL) α-glucosidase inhibition. Retained >60% of inhibitory activity after thermal treatments	[95]
Chlorophyceae				
*Codium isthmocladum*	2 lectins	In vitro, antibiofilm	Binding to monosaccharide residues of the biofilms with preference to α-linkages	[71]
*Halimeda renschii*	Lectin	In vitro, antiviral	High mannose-binding lectin, bonded with Influenza H3N2 viral envelope hemagglutinin, preventing infection	[96]
*Boodlea coacta*	Lectin	In vitro, antiviral	High mannose-binding lectin binds to Influenza hemagglutinin and HIV-1 GP gp120, avoiding infection	[97]
*Caulerpa cupressoides*	Lectin	In vivo, anti-inflammatory and antinociceptive	Analgesic effect as measured by paw licking time. Reduced neutrophil infiltration (65.9%), better than the positive control	[98]
	Lectin	In vivo, anti-inflammatory and antinociceptive	Reduced levels of TNF-α, IL-1β, MPO, and leukocyte infiltration	[99]
	Lectin	In vivo, anti-inflammatory	IL-1β, IL-6, TNF-α and COX-2. Lower neutrophil infiltration	[27]
*Codium decorticatum*	GP	In vitro, antitumor	Apoptosis induction. Mitochondrial membrane alterations, caspase 3 and Bcl-2 genes upregulation	[35]

Notes and abbreviations. GP: Glycoprotein; GPs: glycoproteins: PE: Phycoerythrin; PBP: Phycobiliprotein; DPPH: 2:2-diphenyl-1-picrylhydrazyl; ABTS: 2,2′-azino-bis(3-ethylbenzothiazoline-6-sulfonic acid); HIV: Human immunodeficiency virus; HSV: Herpes simplex virus; SARS-CoV: Severe acute syndrome-coronavirus; IC_50_: Minimum half-inhibitory concentration; IL: Interleukin; TNF-α: Tumor necrosis factor-α; EGFR: Epidermal growth factor receptor; NF-κB: Nuclear factor κb; TLR: Toll-like receptor; ERK: Extracellular receptor kinase; MPO: Myeloperoxidase; MMP: Matrix-metallopeptidase.

### 3.3. Antihypertensive

In the last decade, a wide number of reports have described a hypotensive effect of BAPs from all algae groups. Studies suggest that this effect may be due to an angiotensin I-converter enzyme (ACE) inhibition [26]. Another described mechanism of hypotensive activity is renin inhibition. Renin is an enzyme with various roles, including producing angiotensin I (ACE substrate) and stimulating renal activity [31]. Together, they constitute the known renin-ACE-aldosterone axis, which is the main pathway involved in cardiac pressure regulation [100]. Several studies confirming the anti-hypertensive properties of seaweed BAPs will be mentioned below and have been compiled in Table 3.

A study evaluated the effect of different proteases to obtain a *G. lemaneiformis* protein hydrolysate [52]. The results showed that trypsin produced the extract with the highest ACE inhibitory activity, up to 78%. Further characterization of peptide sequence indicated that QVEY was the major BAP. A similar experiment was carried out with proteins obtained from *U. intestinalis* using Alcalase, α-chymotrypsin, papain, pepsin, and trypsin. Again, trypsin produced the extract with the highest ACE inhibitory activity, and two peptides were indicated as the most active (FGMPLDR and MELVLR) [53]. Another related experiment regarding the use of different proteases to obtain BAPs from *C. lentillifera* indicated that thermolysin was the most appropriate choice in comparison to α-chymotrypsin, pepsin, and trypsin. In this case, the peptides with the highest ACE inhibitory activity were FDGIP and AIDPVRA [101]. Among hydrolytic enzymes, pepsin is one of the most used in the last years. For instance, the peptide sequence NMEKGSSSVVSSRM (with anticoagulant activity) was obtained from the hydrolysis of *N. yezoensis* proteins using this enzyme [56]. A related experiment using *U. rigida* proteins indicated that the peptides with ACE inhibitory activity (IP and AFL) were obtained from the hydrolysis with pepsin [57]. In another experiment with this enzyme, peptides with ACE inhibitory activity (ALLAGDPSVLEDR and VVGGTGPVDEWGIAGAR) were also obtained from *Bangia fusco-purpurea* [102].

Another strategy to obtain hydrolysates from seaweeds is the use of sequential hydrolysis with different enzymes. This strategy was used to obtain peptides with ACE inhibitory activity from *P. palmata* [59]. In this case, the order of hydrolysis was thermolysin, pepsin, trypsin, and chymotrypsin. According to the authors, additional hydrolysis after thermolysin use did not increase the release of peptides with biological potential. Additionally, a reduction in total ACE inhibitory activity in the hydrolysate was obtained after consecutive hydrolysis with thermolysin, pepsin, and trypsin. The peptide sequence LRY was indicated as the most active from the thermolysin hydrolysate. In this sense, it seems reasonable to indicate that the selection of protease and the hydrolysis conditions have a great impact on the release of peptides with biological activity. Nonetheless, additional experiments are required to clarify the role of sequential hydrolysis in the release of bioactive peptides to characterize their protein hydrolyzation patterns and how this affects the properties of yielded BAPs.

The antihypertensive activity of BAPs has also been corroborated in vivo in several studies. For instance, Fitzgerald et al. obtained a renin-inhibitory peptide by hydrolyzing *P. palmata* protein isolates with papain, which sequence was IRLIIVLMPILMA [103]. The peptide showed ACE inhibition at very low concentrations (IC_50_ = 0.32 mg/mL). The same team carried out an in vivo study with this same peptide on spontaneously hypertensive rats but obtained it by chemical synthesis. The effect of the peptide was compared with unpurified *P. palmata* hydrolysate, and the results showed that their effect was very similar, while a significant reduction in systolic blood pressure was achieved [104]. Another in vivo study reported that *U. pinnatifida* BAPs were able to significantly reduce blood pressure in spontaneously hypertensive rats. These BAPs were dipeptides mainly based on Tyr or Trp that showed this hypotensive effect with a single oral dose of 1 mg/kg body weight [105]. To our knowledge, the only clinical trial reporting a reduction in blood pressure was published in 2002 by Shirako Co. Ltd. researchers, which served as a scientific basis for developing and patenting this product. They reported that a 1.8 g/day intake of nori (*Porphyra* sp.) hydrolysate achieved a significant reduction in systolic blood pressure in hypertensive subjects (from 157 to 142 mmHg) but did not in normotensive ones (≤120 mmHg) [106].

**Table 3:** Bioactive peptides and hydrolysates obtained from seaweed proteins.

Species	Hydrolysis	Sequence/s	Bioactivity	Results	Ref.
Rhodophyceae					
*Gracilariopsis lemaneiformis*	Trypsin; E:S (1:25), 37 °C, pH 8, 8 h	QVEY	In vitro, antihypertensive	ACE inhibitory activity. IC_50_= 0.25 mg/mL	[52]
	α-Chymotrypsin; E:S (1:25), 37 °C, pH 8, 2 h	ELWKTF	In vitro, antioxidant	DPPH radical scavenging. EC_50_ = 1.51 mg/mL	[58]
*Porphyra* spp.	Pepsin; E:S (1:100), pH 2, 45 °C, 4 h	GGSK, ELS	In vitro, antidiabetic	α-Amylase inhibition. IC_50_(GGSK) = 0.8 mg/mL, IC_50_(ELS)= 0.9 mg/mL	[54]
*Porphyra dioica*	Alcalase^®^ and Flavourzyme^®^; E:S (1:100), 50 °C, pH 7, 4 h	DYYLR, AGFY, YLVA, AFIT, MKTPITE, TYIA, LDLW	In vitro, antioxidant	Most antioxidant on ORAC assay: IC_50_(AFIT) = 0.4 μg/mL, IC_50_(MKTPITE) = 0.007 mg/mL	[60]
		DYYLR, AGFY, YLVA, TYIA	In vitro, antihypertensive	Most inhibiting BAPs: IC_50_(TYIA) = 0.04 mg/mL, IC_50_(TYIA) = 0.07 mg/mL	[60]
		YLVA	In vitro, antidiabetic	DPP-IV inhibition, IC_50_ = 0.2 mg/mL	[60]
	Prolyve^®^; E:S (1:100), 50 °C, pH 8, 2 h	n.a., increased production of <1 kDa peptides	In vitro, antioxidant	ORAC (IC_50_ = 2.7 mmol TE/g), DPPH (IC_50_ = 0.2 mmol TE/g), FRAP (IC_50_ = 0.4 mmol TE/g)	[107]
*Neopyropia* *yezoensis*	Chemical synthesis	IY, MKY, AKTSY, LRY	Clinical trial, antihypertensive	Blood pression reduction from 157/95 to 142/86 mmHg with 1.8 g/day for 35 days	[106]
	Pepsin; E:S (1:40), pH 2, 45 °C, 2 h	NMEKGSSSVVSSRM	Ex vivo, anticoagulant	Blood clotting retardation; IC_50_ = 4.49 μg/mL	[56]
	Chemical synthesis	“PPY” peptide	In vitro, antitumor	Doses ≥125 ng/mL induced autophagy and apoptosis in MCF-7 cells via the mTOR pathway	[108]
	Chemical synthesis	“PPY” peptide	In vitro, anti-inflammatory	Doses ≥250 ng/mL inhibited expression of inflammatory cytokines in murine macrophages	[29]
*Pyropia* *columbina*	Alkaline protease; E:S (1:40), 55 °C, pH 9.5, 2 h	n.a., ~2.4 kDa peptides	In vitro, antihypertensive	ACE inhibitory activity. IC_50_ = 1.2 mg/mL	[109]
			In vitro, anticoagulant	Antiplatelet aggregation. 2.8 mg/mL achieved 18.7% inhibition	[109]
	Trypsin; E:S (1:20), 50 °C, pH 8, 4 h	n.a., >400 Da peptides	In vitro, antioxidant and anti-inflammatory	DPPH (IC_50_ = 2.8 mg/mL), ABTS (IC_50_ = 2.4 mg/mL); upregulation of IL10 at 0.1 mg/mL	[50]
	Fungal protease; E:S (1:20), 55 °C, pH 4.3, 3 h and Flavourzyme^®^; E:S (1:50), 55 °C, pH 7, 4 h	n.a.	In vitro, anti-inflammatory	Upregulation of IL-10 in murine spenocytes, macrophages and lymphocytes at ≥0.01 mg/mL	[110]
*Palmaria* *palmata*	Chymotrypsin; E:S (1:20), pH 8, 30 °C, 24 h	33 BAPs < 10 kDa	In vitro, antioxidant and antihypertensive	A <10 kDa fraction was the most bioactive at ≥0.75 mg/mL	[55]
	Thermolysin; E:S (1:100), 70 °C, pH 7, 3 h	LRY	In vitro, antihypertensive	ACE inhibitory IC_50_ = 0.01 mg/mL	[59]
	Papain; (20.7 U/ mg protein), 60 °C, pH 6, 24 h	IRLIIVLMPILMA	In vitro, antihypertensive	Renin inhibition. IC_50_ = 0.3 mg/mL	[103]
	Chemical synthesis	IRLIIVLMPILMA	In vivo, antihypertensive	Systolic blood pressure reduction from 187 to 155 mm Hg at 50 mg/kg bw	[104]
*Bangia fusco-purpurea*	Pepsin; E:S (1:200), pH 7.5, 37 °C, 90 min	ALLAGDPSVLEDR, VVGGTGPVDEWGIAGAR	In vitro, antihypertensive	ACE inhibition. IC_50_(ALLAGDPSVLEDR) = 57.2 mg/mL, IC_50_(VVGGTGPVDEWGIAGAR) = 66.2 μg/mL	[102]
Phaeophyceae					
*Saccharina longicruris*	Trypsin; E:S (1:20), pH 7, 30 °C, 24 h	EAESSLTGGNGCAK, IGNGGELPR, ILVLQSNQIR, ISAILPSR, ISGLIYEETR, LPDAALNR, MALSSLPR, QVHPDTGISK, TITLDVEPSDTIDGVK	In vitro, antimicrobial	Mix of all peptides (2.50 mg/mL) inhibited 40% *Staphylococcus aureus* growth	[81]
*Sargassum maclurei*	Pepsin; (80 U/g protein), 37 °C, pH 2, 2 h and Papain; (60 U/g protein), 50 °C, pH 7, 3 h	RWDISQPY	In vivo and in vitro, antihypertensive	Systolic blood pressure reduction from 170 to 150 mm Hg at 100 mg/kg bw; 25% endothelin-1 inhibition at 1.5 mg/mL	[21]
*Laminaria japonica*	Alcalase^®^, papain, trypsin, and pepsin; 55 °C, pH 7.5. E:S and time not specified	KY, GKY, SKTY, AKY, AKY, AKYSY, KKFY, FY, KFKY	In vitro, antihypertensive	ACE inhibition. Hydrolysate of all combined proteases achieved IC_50_ = 0.6 mg/mL	[111]
*Undaria pinnatifida*	Hot water; 93 °C, 20 min	YH, KW, KY, KF, VW, VF, IY, IW, VY	In vivo, antihypertensive	Systolic blood pressure reduction (25–34 mm Hg) by 10 mg/kg bw for 1 week	[112]
	Pepsin; E:S (100:1), 45 °C, pH 2, 18 h	YH, KW, KY, KF, VW, VF, IY, IW, VY	In vivo, antihypertensive	Systolic blood pressure reduction (14–21 mm Hg) by 1 mg/kg bw for 1 week	[105]
	Bromelain; (12 kU/g protein), 45 °C, pH 6, 4 h	KNFL	In vitro, antihypertensive	ACE inhibition. IC_50_ = 1.3 μg/mL	[113]
Chlorophyceae					
*Ulva intestinalis*	Trypsin; E:S (1:25), 37 °C, pH 8, 5 h	FGMPLDR, MELVLR	In vitro, antihypertensive	ACE inhibition. IC_50_(FGMPLDR, MELVLR) = 0.18 mg/mL	[53]
*Ulva rigida*	Pepsin; E:S (1:100), pH 2.0, 37 °C 20 h	IP, AFL	In vitro, antihypertensive	ACE inhibition. IC_50_(IP, AFL) = 0.2 mg/mL	[57]
*Ulva lactuca*	Papain; E:S (1:100), 60 °C, pH 6, 24 h	55 non-allergenic BAPs identified	In vitro, antihypertensive	ACE inhibition (93%) in >1 kDa hydrolysate fraction	[114]
*Enteromorpha clathrata*	Alcalase^®^; 2.9 kU/g protein, T not stated, pH 7.6, 90 min	PAFG	In vitro, antihypertensive	ACE inhibition. IC_50_ = 0.014 mg/mL	[115]
*Caulerpa lentillifera*	Thermolysin; E:S (1:50), 60 °C, pH 8.5, 16 h	FDGIP, AIDPVRA	In vitro, antihypertensive	ACE inhibition. IC_50_ (FDGIP)= 0.03 mg/mL, IC_50_ (AIDPVRA)= 0.04 mg/mL	[101]

Abbreviations: E:S: Enzyme:substrate (*w*/*w*); IC_50_: Half-inhibitory concentration; TE: Trolox equivalents: BAPs: bioactive peptides; ACE: angiotensin I-converter enzyme; DPPH: 2,2-diphenyl-1-picrylhydrazyl; ABTS: (2,2′-azino-bis(3-ethylbenzothiazoline-6-sulfonic acid)); IL: interleukin; ORAC: oxygen radical absorbance capacity; FRAP: ferric reducing ability of plasma; mTOR: mammalian target of rapamycin.

### 3.4. Anti-Inflammatory

Inflammation is a complex response to cell and/or tissue damage, which can be triggered by several factors, such as oxidative damage, infections, or cancer [116]. These processes and cellular interactions involve pro- and anti-inflammatory mediators, which up- or downregulate the inflammatory response. Therefore, the mechanisms by which SP and BAPs have been described to induce anti-inflammatory effects, both in vitro and in vivo, are related to an increased expression of anti-inflammatory mediators and/or downregulation of the expression of pro-inflammatory ones. The main pro-inflammatory mediators are cytokines like interleukins (IL) (e.g., IL-1β, 2, 6, and 8) and also tumor necrosis factor-α (TNF-α). Other compounds like the prostaglandin cyclooxygenase-2 (COX-2) or the chemokine inducible nitric oxide synthase (iNOS) also play a vital role in inducing an inflammatory response, especially producing ROS and nitric oxide (NO), which mainly activate macrophages. These cells, when activated, e.g., via toll-like receptors (TLR), lead these innate immune cells to release IL-6, TNF-α, and also transforming growth factor-β, which promotes cell proliferation [117]. The nuclear factor κB (NF-κB) and mitogen-activated protein kinase (MAPK) are also key pro-inflammatory intermediaries that are produced after TLR activation [118].

Regarding SP with anti-inflammatory activity, most of the studies point to lectins as prime examples. In fact, several in vivo experiments show that these proteins exert anti-inflammatory properties through various pathways. For example, various works have assessed the in vivo anti-inflammatory activity of lectins isolated from the green alga *C. cupressoides* (Table 2). Mice submitted to high concentrations of carrageenan to induce inflammatory response were treated with *C. cupressoides* lectins administered intravenously. The lectin treatment showed similar results to that of dexamethasone, significantly reducing inflammation signals, such as neutrophil levels and paw edema. Additionally, the lectins appeared to reduce nociception while not displaying any significant variation in organs weight or transaminase levels [98]. Very similar results were obtained in rats with temporomandibular induced arthritis. In this study, after the same treatment, the tissue showed a significant decrease in TNF-α, IL-1β, and myeloperoxidase (MPO) levels, as well as a significant reduction in leukocyte concentration [99]. Additionally, it was found that lectin treatment was able to reverse the tissue damage and exert an analgesic effect via a non-opioid pathway. In another work, the authors described the molecular pathway through which the lectin from *C. cupressoides* exerts anti-inflammatory effects, using several pro-inflammatory agents injected in rat paws [27]. A combination of enzymatic and immunohistochemistry staining methods unveiled that this lectin elicited an anti-inflammatory effect by inhibiting the primary cytokines IL-1β, IL-6, TNF-α, and COX-2, as well as lowering neutrophil infiltration, as demonstrated by reduced MPO levels. However, no inhibition of hemeoxygenase-2 (HO-2) was observed. Additionally, the authors confirmed that the anti-inflammatory effect was due to the carbohydrate-binding site of the lectin since when co-administered with mucin, its anti-inflammatory effect was inhibited. This was confirmed by testing the anti-inflammatory effect of the lectin not only against carrageenan but also against dextran and histamine, with successful reductions in paw edemas. However, when directly injected into rat paws instead of intravenously, the lectin induced a strong inflammatory response, which is related to the absent interaction with pro-inflammatory agents but also to the administration route [27]. A 30 kDa lectin purified from *Amansia multifida* displayed similar effects, modulating paw edema and peritonitis formation in mice [87]. This lectin consistently ameliorated paw edema against carrageenan, histamine, prostaglandin E2, and compound 48/80 (an inducer of histamine release). Pre-treatment with this lectin also greatly reduced neutrophil infiltration following carrageenan-induced peritonitis, as well as significantly lower TNF-α IL-1β expression in the affected tissue (Table 2). Moreover, its administration showed lower MPO activity, with values even lower than those observed for indomethacin. Its anti-inflammatory effect was also confirmed by the increased levels of glutathione, which were greater than in the control group, thus indicating that its anti-inflammatory property is also related to preventing further oxidative damage [87].

Other studies have reported the anti-inflammatory properties of other SP, although few examples can be found. For instance, a 3.5 kDa GP from *N. yezoensis* was tested in vitro lipopolysaccharide (LPS)-activated murine RAW 264.7 macrophages [86]. This GP reduced IL-1 receptor-associated kinase 4 binding to the macrophages’ TLR4, which inhibited NF-κB and resulted in lower production of TNF-α, IL-1β, COX-2, and iNOS. Additionally, phosphorylation of Jun N-terminal kinase (JNK) and extracellular signal-related kinase (ERK) was lowered, which are also relevant mediators to the MAPK inflammatory pathway [86].

BAPs have also been reported to ameliorate inflammatory responses, but only in vitro. For instance, *P. columbina* hydrolysates managed to induce anti-inflammatory responses [110]. Using macrophages, T lymphocytes, and splenocytes isolated from rat spleen, cell toxicity, proliferation, and cytokine production were assessed. The authors found that the hydrolysate showed no toxicity, and, in combination with concanavalin A (a lymphocyte mitogen), it exerted a synergistic effect on lymphocyte proliferation. The hydrolysate also decreased TNF-α and IL-6 while increasing IL-10 release. The observed anti-inflammatory effect was, therefore, due to an increased IL-10 release, as this interleukin acts as an anti-inflammatory mediator in macrophages and lymphocytes while suppressing the MAPK and NF-κB dependent pathways. The same team tested the anti-inflammatory potential of an SP hydrolysate obtained from the simultaneous hydrolyzation of various *Ulva* species [62]. Employing the same experimental model as in the previous work, the anti-inflammatory pathways of this hydrolysate were tested. These hydrolysates showed an anti-inflammatory but also an immunomodulatory effect [62]. Similarly, in vitro anti-inflammatory potential of the BAP PPY1 from *N. yezoensis* was tested. Using LPS-stimulated RAW 264.7 macrophages, the authors found reduced expression of iNOS, COX-2, IL-1β, and NF-κB. This downregulation of pro-inflammatory mediators was linked to lower expression of p38 mitogen-activated protein kinases and JNK [29].

### 3.5. Antitumoral

Cancer is a complex disease characterized by uncontrolled cell proliferation. Inflammation, oxidative stress, and immunomodulation have interconnected roles on cancer as inducer factors but also as outcomes of it [119]. Among several cancer treatment options, cytostatic agents or specific antitumor molecules are the focus of novel anticancer drugs [120]. Although there are several pathways resulting in cell apoptosis, the major mechanisms are regulated by caspase proteins, the mammalian target of rapamycin (mTOR), and others, like up- or downregulation of Bcl-2 family genes or inhibition of protein-53. In this sense, several SP and BAPs have been described to exert antitumoral effects on various in vitro models. However, in vivo studies are still necessary to assess their potential.

Lectins play a role in immunology by agglutinating the carbohydrate domain of several membrane GPs. This allows seaweed mannose-specific lectins to bind to specific GPs from certain tumoral cells and block their proliferation. Although reports on specific anticancer studies testing seaweed lectins are scarce, a recent study reported anticancer properties from two *S. filiformis* lectins [89]. Testing on human breast (MCF-7) cancer cells, it was found that these lectins induced apoptosis, relating to downregulation of antiapoptotic genes like Bcl-2 and upregulation of proapoptotic genes like Bax and caspases 3, 8, and 9. The authors determined that this anticancer activity could also be due to the mannose oligosaccharides present in the MCF-7 cells surface, for which these lectins were specific [89]. Another study reported that PE from *G. lemaneiformis* induced apoptosis in human colon (SW480) cancer cells. Following a thorough proteomic and morphological analysis, exposition to PE resulted in a loss of cell adherence, arresting the cell cycle at S and G_2_/M phases, and inducing apoptosis. Apoptosis was evidenced by flow cytometry but also by significant expression of the apoptotic genes for caspase-9 and 3 and protein-53. The loss in cell adherence, besides observed cells, was demonstrated by a lower expression of annexin A2, which resulted in lower cell adherence and is also related to an upregulation of caspase-3 or protein-53 [90]. Similarly, R-PE from *Portiera hornemannii* was tested against hepatic (HepG2) and lung (A549) carcinoma cells. Their results indicated that this R-PE induced apoptosis in these cancer cell lines by arresting the cell cycle at the G_2_/M phase, reducing cell proliferation, and leading to DNA fragmentation. This was demonstrated by fluorescence microscopy, which evidenced that this response was dose-dependent [91]. MAAs have also been described to possess antitumoral properties to a certain degree, possibly because of their potent antioxidant activity. Various MAA-rich extracts from *C. chrispus*, *M. estellatus*, and *P. palmata* were tested for their anticancer activity against cervical (HeLa) cancer and lymphoma (U937) cells [121]. The study determined that these extracts increased the activity of caspases 3 and 7, which was related to changes in cells to apoptotic morphology.

Regarding BAPs, few studies have corroborated their antitumoral properties. For example, the peptide PPY from *N. yezoensis* was reported to induce apoptosis in MCF-7 cancer cells. The study focused on the PPY potential to inhibit the insulin-like growth factor-I receptor (IGF-IR), as its overexpression is related to tumor proliferation and survival. After fluorescent staining, a reduction in cell proliferation and increased apoptosis was observed. The underlying molecular mechanism was related to a decreased activation of p85, which is a subunit of phosphatidylinositol 3-kinases (PI3K) and is a critical regulator of cell proliferation and differentiation. Additionally, IGF-R expression was significantly reduced, as well as ERK, which decreased inflammation and induced apoptosis. Cell cycle arrest was evidenced by cyclins down-expression, which was correlated with the cyclin inhibitors p21 and p27 [122]. The same team later described that these anticancer effects of PPY were also related to an upregulation of mTOR, among other key apoptosis inducers. Additionally, these MCF-7 cells also showed downregulation of p70S6 kinase, the expression of which is related to cancer metastasis [108].

### 3.6. Anti-Diabetic and Anti-Obesity

Type 2 diabetes mellitus and obesity are metabolic syndromes developed by hormonal and/or diet imbalances that may derive from several related diseases. To prevent or ameliorate these diseases, there are several known target molecules, such as dipeptidyl peptidase-IV (DPP-IV) and the digestive enzymes α-glucosidase and α-amylase. DPP-IV is a transmembrane exopeptidase enzyme that degrades proteinic hormones, such as the glucagon-like peptide-1 (GLP-1). Both α-glucosidase and α-glucosidase metabolize glucose and starch, respectively, in the small intestine [123]. GLP-1 is an incretin hormone that induces the secretion of glucose-dependent insulin, as well as producing a satiety sensation [124]. Therefore, inhibiting the action of these mentioned enzymes and promoting secretion of both GLP-1 and insulin would result in improved management of total glucose plasma levels and metabolic function.

According to the literature, most of the studies reporting anti-diabetic and anti-obesity properties of seaweed compounds are related to BAPs. For instance, BAPs from *N. haitanensis*, obtained by ultrasound extraction and simulated gastric digestion, showed α-glucosidase inhibitory effects. The peptides were separated into several fractions by gel chromatography, and those with higher peptide content and lower MW displayed more antioxidant and inhibitory activities [68]. Similarly, BAPs from a <3 kDa *Porphyra* spp. hydrolysate fraction displayed very high α-amylase-inhibitory activity. The inhibitory activity was confirmed by artificial synthesis of the peptides, achieving inhibition of IC_50_ = 2.58 mM. Further isolation of peptides revealed that GGSK and ELS were the most relevant BAPs [54]. As noted by the authors, this inhibition mechanism was not only related to the lower MW of the peptides but also that it was non-competitive with the substrate. Several studies have also shown the inhibitory activity against DPP-IV of *P. palmata* SP hydrolysates and BAPs. An SP hydrolysate from this alga significantly inhibited DPP-IV, with an IC_50_ of 1.17 mg/mL, which was a quarter of the concentration needed for the untreated protein isolate to achieve the same inhibition rate [125]. Similar results were reported for other hydrolysates obtained with different proteases. Hydrolysate produced with Corolase^®^ displayed the most significant inhibition (IC_50_ = 1.65 mg/mL). Interestingly, the hydrolysate obtained from the aqueous-soluble fraction was more active (IC_50_ = 3.16 mg/mL) than that of the alkaline-soluble one, and the combination of both fractions showed an intermediate activity (IC_50_ = 2.26 mg/mL) [126]. Several BAPs from *P. palmata* hydrolysate with DDP-IV, ACE inhibition, and antioxidant activities were identified. The authors cross-checked these isolated peptide sequences and found that many of these were part of PBPs and the RuBisCo large subunit, which indicates that these proteins may contain the most bioactive peptide sequences [60]. Finally, it has been reported that a BAP from *N. yezoensis* stimulated the proliferation and differentiation of murine small intestine cells [127]. This was achieved at concentrations from 1 μg/mL by triggering the IGF-IR signaling pathway, which induces diverse changes in key protein expression in the cell nucleus and leads to cell differentiation. The authors analyzed that this occurred via upregulation of this receptor’s substrates such as IGF-IR, insulin receptor substrate-I (IRS-I), sarcoma homology collagen, and phosphotyrosine. This upregulation inducing an increased response of IGF-IR leads to the activation of other related pathways that result in tissue development and angiogenesis without increasing an inflammatory response. This effect of *N. yezoensis* BAP may be accounted for a functional role in small intestinal epithelial tissue development [127].

Anti-diabetic and anti-obesity effects of seaweed BAPs have also been corroborated by in vivo studies. A study reported that oral treatment for 18 days with *P. palmata* hydrolysate to diabetic mice resulted in a significant reduction in glycemia, increased insulin secretion, and reduced glycated hemoglobin levels. Moreover, this protein hydrolysate also reduced energy intake and increased total plasma GLP-1 levels. The most relevant highlight of the study was that these responses were not statistically different from those of the positive control (metformin) under the same treatment conditions [128]. The same team assessed several insulinotropic and toxicity effects of a *P. palmata* hydrolysate digested with different proteases and simulated gastric digestion. Using various suitable pancreatic, adipose and enteroendocrine cell cultures, they found that not only the hydrolysates did inhibit DPP-IV, but also increased insulin and GLP-1 secretion. The authors inferred that this was due to an actual response and not the release of these molecules following cell death. An increase in calcium transmembrane mobilization, as well as an upregulation of cyclic adenosine monophosphate (cAMP) was found liable for the overall incretin effect. When testing these effects in vivo with mice (100 mg/kg body weight), it was found an improved glucose tolerance, as well as satiety response after 90 min of oral administration, which would indicate a positive increase in GLP-1 and other incretin upregulation [129].

## 4. Potential Applications

### 4.1. Seaweed Proteins

**Figure 4:**
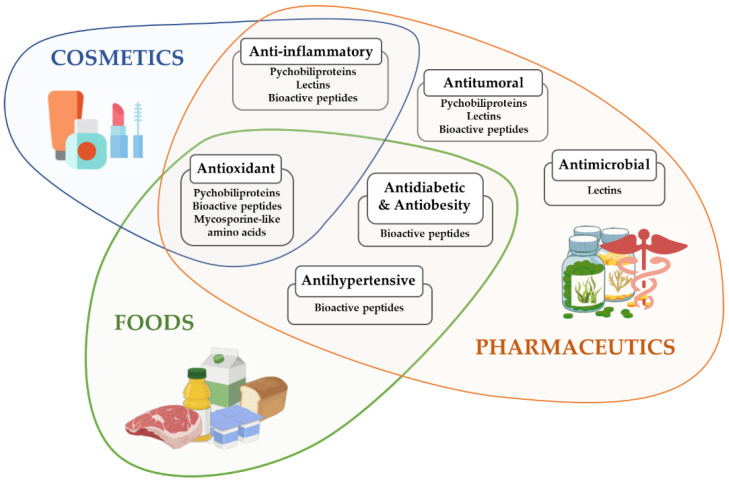
Potential applications of seaweed proteins, bioactive peptides, and Mycosporine-like amino acids considering their described bioactivities. Several properties of these molecules may be applied to diverse fields and industries in different formulations.

Considering the described biological properties of SPs, several potential applications have been proposed. One of the most promising SP groups is lectins due to their broad range of bioactivities described. Based on their antimicrobial and antiviral properties, seaweed lectins could be considered as new ingredients for the formulation of treatments against some pathogens. Indeed, as mentioned before, the lectin griffithsin has been described to be able to treat and prevent infections in both rats and macaques, specifically of Influenza, HSV, and HIV [75]. Topical application of this lectin in pharmaceutical formulations could prove to be a possible preventive treatment against these infections, as evidenced by multiple reports. Moreover, seaweed lectins from diverse sources have shown to be effective at pico- or nanomolar concentrations for exerting either antimicrobial, anti-inflammatory, or antitumor effects (Figure 4). In this last regard, many studies have assessed the potential cytotoxicity of these lectins, finding very low cytotoxicity indexes or even no binding with healthy cells, in contrast to tumoral ones [92]. The anti-inflammatory effect of different lectins also supports potential pharmaceutical applications, especially in the case of *C. cupressoides* lectin, as it displays in vivo analgesic effects [27].

Regarding PBPs and MAAs, they could also be considered useful compounds for further industrial applications. PBPs, such as PE, are currently used as fluorescent probes in biochemistry, but their antioxidant, anti-inflammatory, and antitumoral properties suggest further applications. Indeed, they can be used as colorants in foods, as well as functional ingredients, based on their antioxidant properties. For instance, this was tested in edible films with incorporated *P. columbina* R-PE, which displayed antioxidant activity [130]. On the other hand, MAAs are excellent antioxidant molecules, which, paired with their very low cytotoxicity and high thermal resistance, make them ideal food additives [121]. Considering that MAAs seem to play a specific role as UV oxidation protectors in seaweeds, these could also be included in cosmetics such as sunscreens [131].

### 4.2. Bioactive Peptides

Scientific evidence supports the biological effects of seaweed BAPs (Table 3), as it has also been reported with BAPs from other protein sources [132,133]. The main biological effects reported are related to the improvement of hypertension, often described by inhibiting ACE or renin activities [52,101,126]. Given that this effect has been verified in various in vivo experiments [21,112] and also reported in human subjects [106], there is a scientific basis to suggest that seaweed BAPs should be considered as cardioprotective agents. Additionally, some BAPs from *N. yezoensis* were reported to exert anticoagulant activity, which would contribute to cardiac function in some cases [56].

Seaweed BAPs have also been described to be potent antioxidant molecules at low concentrations and in various antioxidant assays [107]. This suggests that their use as a functional ingredient in foods or cosmetics could be multivalent, acting as preservers and/or dietary antioxidant compounds.

Furthermore, BAPs, such as described from *P. palmata*, are reported to exert antidiabetic and anti-obesity effects [128]. Foremost, these effects are related to upregulation of GLP-1 and/or inhibition of DPP-IV, which results in an improved metabolic function and control of glucose levels [60]. Seaweed BAPs may also act as α-glucosidase inhibitors, reducing gut glucose absorption and subsequent glycemic levels [54]. Given that these effects have been described in animal models, there is a great potential for these peptides as dietary supplements for diabetes and/or obesity management [128,129]. The anti-inflammatory or antitumoral properties of seaweed BAPs have also been highlighted. Anti-inflammatory effects of seaweed BAPs are mostly related to the upregulation of anti-inflammatory cytokines such as IL10, ameliorating inflammatory reactions [110], but also through immunomodulation [62]. Regarding antitumoral effects, while available reports have only been performed in vitro, several works describe these peptides to induce apoptosis while showing almost null cytotoxicity [108,134]. However, while there is abundant evidence of in vitro describing molecular effects of these BAPs, more animal studies are required to validate and corroborate these properties to devise effective applications. Notably, the great majority of seaweed BAPs are of short length, which has proven to make them resistant to gastrointestinal digestions and retain their biological properties, such as antihypertensive or antioxidant [104,105]. This fact further supports their suitability as food additives or dietary supplements.

Altogether, even though accumulating reports support the bioactive properties of seaweed BAPs, additional studies are necessary to improve the knowledge about the use of seaweed protein hydrolysates and bioactive peptides in different systems and fields of research. Further research should focus on deeper studies about effects in vivo and direct applications in (i.e., pharmacological, food science, and cosmetics) to explore the wide variety of activities of seaweed peptides.

## 5. Conclusions

Seaweeds are being increasingly exploited on a global scale due to their dietary and nutritional benefits, of which their protein and amino acid composition pose a valuable component. Considering their protein content and bioactivities, seaweeds can be feasible to use as a source of these compounds with functional properties. SP such as red algae’s PBPs, as well as lectins, have been extensively studied in recent years as potential new functional ingredients or pharmacological agents since significant evidence supports their use as anti-inflammatory, antitumoral, or antiviral agents. Moreover, SP hydrolysates contain several BAPs, which have been isolated and demonstrated to possess effective biological properties such as the ones mentioned above but also to be anti-diabetic or anti-obesity agents, as well as important hypotensive molecules. Several works have analyzed the potential mechanisms of action of these proteins and derived peptides, highlighting their effective potential for various applications. Altogether, despite the growing evidence on SP and their derived BAPs, much further work is needed to evaluate the extent of their possible mechanisms of action. Likewise, further works would need to assess potential bioactive SP in many species, especially from green or brown algae, as these are less studied groups.

## Abbreviations

SP	Seaweed proteins
AA	Amino acids
EAA	Essential amino acids
NEAA	Non-essential amino acids
BAPs	Bioactive peptides
GP	Glycoprotein
PBP	Phycobiliprotein
PE	Phycoerythrin
MAAs	Mycosporine-like amino acids
λ_max_	Absorption maxima
ORAC	Oxygen radical absorption capacity
FRAP	Ferric reducing antioxidant power
DPPH	2,2-diphenyl-1-picrylhydrazyl
ABTS	2,2′-azino-bis(3-ethylbenzothiazoline-6-sulfonic acid)
UV	Ultraviolet
ACE	Angiotensin-I converter enzyme
ROS	Reactive oxygen species
HIV	Human immunodeficiency virus
HSV	Herpes simplex virus
HPV	Human papilloma virus
SARS-CoV	Severe acute respiratory syndrome-coronavirus
IL	Interleukin
TNF-α	Tumor necrosis factor-α
COX-2	Cyclooxygenase-2
NO	Nitric oxide synthase
iNOS	Nitric oxide
TLR	Toll-like receptors
TGF-β	Transforming growth factor-β
NF-κB	Nuclear factor κb
MAPK	Mitogen-activated protein kinase
LPS	Lipopolysaccharides
MPO	Myeloperoxidase
HO-2	Hemeoxygenase-2
JNK	Jun N-terminal kinase
ERK	Extracellular signal-related kinase
mTOR	Mammalian target of rapamycin
IGF-IR	Insulin-like growth factor-I receptor
PI3K	Phosphatidylinositol 3-kinase
DPP-IV	Dipeptidyl peptidase-IV
GLP-1	Glucagon-like peptide-1
IRS-I	Insulin receptor substrate-I
